# The Development of Dry Eye Disease after Surgery-Indicated Chronic Rhinosinusitis: A Population-Based Cohort Study

**DOI:** 10.3390/ijerph17113829

**Published:** 2020-05-28

**Authors:** Chia-Yi Lee, Kun-Lin Yang, Chi-Chin Sun, Jing-Yang Huang, Hung-Chih Chen, Hung-Chi Chen, Shun-Fa Yang

**Affiliations:** 1 Department of Ophthalmology, Show Chwan Memorial Hospital, Changhua 50093, Taiwan; ao6u.3msn@hotmail.com (C.-Y.L.); b101098004@tmu.edu.tw (H.-C.C.); 2 Department of Optometry, College of Medicine and Life Science, Chung Hwa University of Medical Technology, Tainan 717, Taiwan; 3 Department of Otolaryngology–Head and Neck Surgery, Kaohsiung Chang Gung Memorial Hospital, Kaohsiung 833, Taiwan; mp9371@cgmh.org.tw; 4 Department of Ophthalmology, Chang Gung Memorial Hospital, Keelung 20402, Taiwan; arvin.sun@msa.hinet.net; 5 Department of Chinese Medicine, Chang Gung University, Taoyuan City 33302, Taiwan; 6 Department of Medical Research, Chung Shan Medical University Hospital, Taichung 40201, Taiwan; wchinyang@gmail.com; 7 Department of Ophthalmology, Chang Gung Memorial Hospital, Linkou 33305, Taiwan; 8 Department of Medicine, Chang Gung University College of Medicine, Taoyuan 33302, Taiwan; 9 Center for Tissue Engineering, Chang Gung Memorial Hospital, Linkou 33305, Taiwan; 10 Institute of Medicine, Chung Shan Medical University, Taichung 40201, Taiwan

**Keywords:** chronic rhinosinusitis, functional endoscopic sinus surgery, dry eye disease, ocular surface, population-based

## Abstract

We aim to evaluate the risk of dry eye disease (DED) occurrence in patients with surgery-indicated chronic rhinosinusitis (CRS) via the national health insurance research database in Taiwan. After exclusion, patients with a diagnostic code of CRS and had received functional endoscopic sinus surgery (FESS) were regarded as having surgery-indicated CRS and enrolled in the study group, then each patient in the study group was age- and gender-matched to four non-CRS patients that served as the control group. The outcome was considered as the development of DED and Cox proportional hazard regression was used for the statistical analysis, which involved multiple potential risk factors of DED. A total of 6076 patients with surgery-indicated CRS that received FESS and another 24,304 non-CRS individuals were enrolled after exclusion. There were 317 and 770 DED events in the study group and the control group during the 16-year follow-up interval, and the study group demonstrated a significantly higher adjusted hazard ratio (1490, 95% confidence intervals (CI): 1.303-1.702) of DED development compared to the control group in the multivariable analysis. In addition, the cumulative probability analysis illustrated a positive correlation of DED occurrence and the disease period of surgery-indicated CRS (*p* < 0.0001). In the subgroup analysis, both genders revealed a higher but not significant incidence of developing DED in the study group. In conclusion, the existence of surgery-indicated CRS will increase the risk of developing DED, which correlated to the disease interval.

## 1. Introduction

Chronic rhinosinusitis (CRS) refers to inflammation in the paranasal sinuses that persists for at least three months [1], and affects approximately 6% of the population [2]. The clinical presentations of CRS include nasal stiffness, nasal discharge, facial pain, reduction of smell, headache and shortness of breath [1,3]. Except for the symptoms mentioned above, CRS may also be associated with other inflammatory disorders like allergic rhinitis and asthma [4]. In the severe form, the infection and inflammation of the paranasal sinus in CRS may even lead to the occurrence of fatal intracranial infection including brain abscesses [5]. 

Both medical and surgical approaches have been utilized to treat CRS [6]. The local corticosteroid therapy, systemic corticosteroid usage and antibiotic treatment have been used to treat CRS with favorable outcomes [3,6]. In addition, functional endoscopic sinus surgery (FESS) is a well-established procedure for severe CRS that shows poor response to medical management and can yield high anatomical success rate after the surgery [7,8,9]. Still, the recovery of maxillary sinus mucosa in patient with CRS is incomplete one year after the FESS performance [3]. Moreover, patients with certain risk factors, like higher Lund-Mackay CT scores and those with fungal-induced CRS, may still experience a poor quality of life or persistent nasal polyp formation even after successful FESS intervention [7,8]. The above lines of evidence suggest that the local effect of severe CRS would endure despite the FESS management.

Concerning the ocular complications related to the development of CRS, orbital cellulitis, preseptal cellulitis and subperiosteal abscess have been reported in a previous cross-sectional study [9]. In another report, inflammatory conjunctivitis and scleritis were observed in a patient with right maxillary sinusitis [10]. Symptoms of dry eye disease (DED) can include a sensation of dryness, irritation resulting from evaporative excess, aqueous deficiency, ocular surface damage, or all of these symptoms [11]. Recently, the major pathophysiology of DED is speculated as a multi-factorial vicious cycle that leads to inflammatory processes [12]. Since the etiology of CRS is usually related to inflammation [13], a certain association may exist between these two disorders, which has rarely been reported elsewhere.

Herein, we aim to investigate the relationship between surgery-indicated CRS and the development of DED via the National Health Insurance Research Database (NHIRD) in Taiwan. Additionally, we also conducted a multivariate model to estimate several risk factors of DED.

## 2. Materials and Methods 

### 2.1. Ethics Declaration and Data Resource

This retrospective, population-based cohort study adhered to the Declaration of Helsinki in 1964 and its late amendment. In addition, the current study was approved by both the National Health Insurance Administration and the Institutional Review Board of Chung Shan Medical University, Taichung, Taiwan. In addition, the need for informed consent was waived by the above two institutions. The claims data used in the current study were obtained from the Longitudinal Health Insurance Database 2005 version (LHID), which derived the data from the NHIRD that contains data of insurance claims from more than 99% of Taiwan’s population. The data of LHID was randomly sampled from the NHIRD registry for the year 2005 by the database of the National Health Insurance Administration. The information/resource available from the LHID included the demographic data of the subjects, their socioeconomic conditions, the residence of the subjects, the International Classification of Diseases-Ninth Revision (ICD-9), the International Classification of Diseases-Tenth Revision (ICD-10), and the medications used for the study subjects. The time interval of LHID ranges from 1 January 2000 to 31 December 2016, with a total study interval of about 16 years.

### 2.2. Patient Selection

Patients were set as having surgery-indicated CRS if the following criteria was accomplished (1) in receipt of the diagnosis of CRS, (2) the arrangement of FESS within two year after the diagnosis of CRS, (3) the use of corticosteroid or antibiotics for at least two years from the diagnosis of CRS and (4) receipt of the CRS diagnosis by an otorhinolaryngologist. The index date was defined as the date of two years after the diagnosis of surgery-indicated CRS. In addition, the following exclusion criteria were applied to exclude certain impaired ocular conditions: (1) receipt of a diagnosis of legal blindness at any time; (2) receipt a diagnosis of ophthalmic tumors at any time; (3) receipt of a diagnosis of severe ocular trauma at any time; (4) receipt of eyeball removal surgery before the index date; and (5) receipt a diagnosis of DED (diagnostic codes are listed in the next paragraph) before the index date. In addition, each individual in the study group was age and gender-matched to four non-CRS subjects, as discussed in the following sections, which served the control group. Still, individuals with surgery-indicated CRS who could not be matched with four non-CRS patients were excluded from the current study.

### 2.3. Main Outcome Measurement

The development of DED was defined as the main outcome in the current study, which was based on the DED-related diagnostic codes after the index date. In clinical practice, ICD-9/ICD-10 codes for “unspecific corneal disorder” may also be used for some forms of DED, but these ICD-9/ICD-10 codes were eliminated to prevent overestimation and confusion of the primary outcome. Moreover, only those DED patients diagnosed by an ophthalmologist were considered as having achieved the primary outcome and included in the current study.

### 2.4. Demographic Variables and Co-Morbidities

To standardize the health condition of participants, we also considered the effects of demographic conditions including, age, gender, and income level and the following co-morbidities in the analysis: hypertension, diabetes mellitus, ischemic heart diseases, hyperlipidemia, congestive heart failure, peripheral vascular disease, cerebrovascular disease, dementia, chronic pulmonary disease and asthma, rheumatic disease, peptic ulcer disease, liver disease, and hemiplegia/paraplegia. To make the ocular condition of the study population more homogenous, we also included the effect of keratopathy, uveitis, glaucoma, and age-related macular degeneration (AMD) in the multivariable model. We would then longitudinally follow the patients’ condition from the index date until the date of any type of DED diagnosis, or until the last date of data collection from the LHID, which means 31 December 2016.

### 2.5. Statistical Analysis

SAS version 9.4 (SAS Institute Inc., NC, USA) was employed for all the statistical analyses in the current study. After the age and gender-matching of the study group and the control group with a 1:4 ratios, the incidence rate ratio, crude relative risk and corresponding 95% confidence intervals (CI) were calculated by the Poisson regression. Then, the Cox proportional hazard regression was adopted to yield adjusted hazard ratios (aHR) of DED by incorporating the above demographic data, ocular diseases and systemic comorbidities in the multivariable analysis. In addition, the Kaplan–Meier curves were plotted to show the cumulative probability of DED between the study and control groups, and we applied the log-rank test to evaluate the significant difference between the two survival curves. For the subgroup analysis, the sensitivity analysis with aHR of DED that stratified by the gender and age subgroups were conducted. Because most individuals in the LHID/NHIRD are Han Taiwanese, race was not considered a covariate. Statistical significance was set at *p* < 0.05. 

## 3. Results

**Figure 1:**
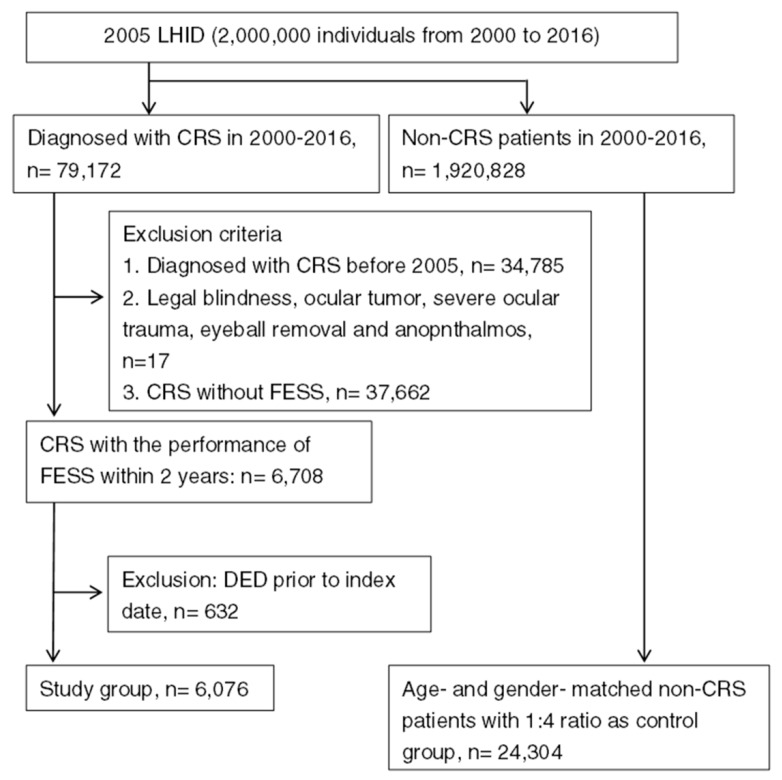
The flowchart of patient selection. LHID 2005: Longitudinal Health Insurance Database 2005 version, CRS: chronic rhinosinusitis, FESS: functional endoscopic sinus surgery, DED: dry eye disease.

A total numbers of 6076 patients with surgery-indicated CRS were enrolled in the study group, while another 24,304 individuals were enrolled into the control group after exclusion, and the flowchart of patient selection is shown in Figure 1. The age and gender ratio are identical due to the matching process, while the different characteristics of co-morbidities between the study and control group are listed in Table 1.

**Figure 2:**
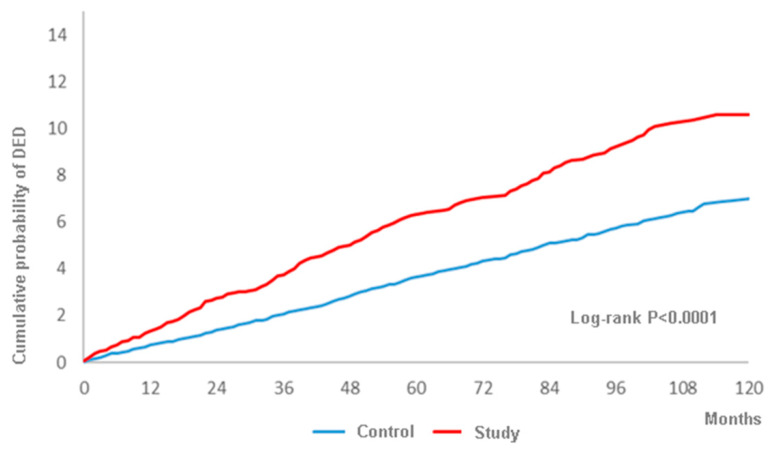
The cumulative probability of dry eye disease between the study and control groups.

A total of 317 and 770 DED events were observed in the study group and the control group during the whole follow-up period up to 16 years, and the study group showed a higher crude relative risk (1685, 95% CI: 1.479-1.921), as shown in Table 2. Moreover, the study group revealed a significantly higher aHR (1490, 95% CI: 1.303-1.702) compared to the control group after adjusting for multiple potential risk factors including demographic data, systemic diseases and ocular diseases, as shown in Table 3. Besides, the cumulative probability of DED was also significantly higher in the study group according to the result of the log-rank test (*p* < 0.0001) (Figure 2). In addition to surgery-indicated CRS, peripheral vascular disease, chronic pulmonary diseases, peptic ulcer disease, liver disease, keratopathy, uveitis, glaucoma and AMD were prominently related to the higher rate of DED development (Table 3). About the subgroup analysis, both the male and female population in the surgery-indicated CRS yielded a significant higher aHR of developing DED and the aHR of DED increased with older age (Table 4).

## 4. Discussion

In the current study, the occurrence of recent-onset DED is significantly higher in those with surgery-indicated CRS compared to non-CRS individuals after adjusting for multiple potential risk factors in the multivariate analysis. On the other hand, the chance of developing DED is also significantly raised in those individuals diagnosed with peripheral vascular disease, chronic pulmonary diseases, peptic ulcer disease, liver disease, keratopathy, uveitis, glaucoma and AMD.

About the possible pathophysiology between CRS and DED, several mechanisms may lead to such a relationship. Recently, CRS has been found to be an inflammatory disorder and tissue-deforming process rather than an infectious lesion in the majority of cases [4,14,15]. The interleukin is elevated in patients with CRS [16], with raised matrix metalloproteinases detected [17]. In addition, tissue from patients with CRS demonstrated inflammatory cell infiltration, thus suggesting an inflammatory nature of CRS [18]. On the other hand, the vicious cycle in developing DED includes components such as meibomian gland dysfunction (MGD), aqueous deficiency, exposure-related damage and goblet cell deficiency [12]. Among them, both the MGD and goblet cell deficiency are related to the increment inflammatory reaction [12,19], in which MGD will lead to the evaporative excess subtype of DED and affects about 50 percent of patients with DED [20]. Besides, several inflammatory mediators—like interleukin and matrix metalloproteinases—are elevated in those individuals with DED [12,21]. As a consequence, the persistent inflammatory process in prolonged CRS may activate the inflammatory reaction in the DED cycle. In addition, both CRS and DED may manifest vasculitis concurrently [22,23], and thus presence of CRS and underlying vasculitis may be related to the occurrence of DED. Moreover, severe CRS may lead to poor sleep quality [24], which is also a significant risk factor for the formation of DED [25]. Accordingly, persistent and severe CRS may contribute to the development of DED, as demonstrated in the current study.

Concerning the relationship between CRS and DED, one study found that DED would exist in patients with CRS [26]. In the current study, patients with surgery-indicated CRS showed a higher aHR of developing DED (1.490) compared to those without CRS after adjusting several potential risk factors in a multivariate analysis. To our knowledge, this is a preliminary experience to reveal the association between these two diseases. Additionally, we excluded those with preceding DED before the index date (i.e., two years after the diagnosis of CRS), thus the chance of enrolling previous DED cases and mistaking them as the effect of CRS is minimal. Moreover, the log-rank test illustrated a positive and significantly cumulative probability of DED occurring, which was correlated to the disease interval of surgery-indicated CRS. This finding further demonstrates that the effect of surgery-indicated CRS to induce DED is a long-term influence rather than an acute stress result from the FESS. As a consequence, we speculate a causal relationship between surgery-indicated CRS and the development of DED.

In the field of epidemiology, prevalence of DED ranges from 5 to 50 percent [20]. In the current study, the occurrence rate is 14.15% in the study group, which is significantly higher than the occurrence rate in the control group and numerically higher than another epidemiologic study operated in the same region [27]. The finding implies that surgery-indicated CRS not only leads to a higher rate of DED, but also influences a majority of the population with surgery-indicated CRS. Furthermore, CRS is also a prominent disease that affects up to 5 percent of the population, thus decreasing their quality of life [4]. Since both CRS and DED may influence a large number of patients, a referral to the ophthalmic department for to be screened for possible DED may be suggested in patients with long-lasting CRS. On the other hand, the patients with DED and nasal discomfort may also be referred to the otorhinolaryngologic department for a preliminary survey of CRS.

Other diseases that correlated to the occurrence of DED, including keratopathy, uveitis, glaucoma and AMD, showed a significant correlation to DED in the current study. Because the persistent DED may cause damage in the ocular surface including the cornea [12], it is reasonable for the correlation between the two diseases in the current study. In addition, the preservatives in anti-glaucomatous medications may cause further damage of ocular surface and lead to inflammation of the ocular surface and DED [28]. Thus an association between glaucoma and DED is expectable. The uveitis is also an inflammatory disease like DED [29], and age is risk factor for both AMD and DED, which could explain such a correlation [20,30]. The systemic diseases related to DED include peripheral vascular disease, chronic pulmonary diseases, peptic ulcer disease and liver disease, while the exact etiology needs further investigation. Both genders in the study group showed a higher probability to develop DED, while the female gender revealed a numerically higher aHR than the males, which corresponded to the previous epidemiological study [27]. Although older age is a risk factor of DED [20], the bias may be minimized due to our age-matched manipulation.

There are some limitations in the current study. First, a group with patients diagnosed with CRS but without the arrangement of FESS should be included to make the statistical analysis more complete, but this could not be conducted due to the overestimation rate of CRS, which may lead to some confusion [31,32,33,34]. In addition, the retrospective and observational nature of study design might reduce the homogeneity of the patient population even after multivariate analysis. Notwithstanding, we used claimed data rather than real medical documents, thus missing some important information like the laterality of DED and the postoperative condition of CRS after a FESS procedure. In addition, seasonal allergies, in which the DED could be a surrogate for allergic disease in CRS subjects, cannot be included as a potential confounder for the same reason that real medical documents were not available in the current study. Moreover, the usage of cyclosporine emulsion (not reimbursed by the health insurance until the end of 2013) could not be accessed in the majority of the study population, thus it was hard to investigate the percentage of severe DED. Last but not least, only the DED diagnosed by an ophthalmologist was enrolled in the current study to gain the accuracy of DED diagnosis, while some general physicians may also handle a number of DED individuals; thus, the DED population in the current study may be underestimated.

## 5. Conclusions

In conclusion, the presence of surgery-indicated CRS contributes to the development of DED after adjusting for multiple potential risk factors. Furthermore, the occurrence of DED is correlated to the disease interval of surgery-indicated CRS. Further large-scale prospective study to evaluate the effect of CRS on the severity of DED is warranted.

## Figures and Tables

**Table 1:** Different characteristics of co-morbidities between the study and control group.

Baseline Characteristics	Study *N* = 6076	Control *N* = 24,304	*p* Value
Age			1.0000
<40	2283 (37.57%)	9132 (37.57%)	
40–59	2637 (43.4%)	10,548 (43.4%)	
60–79	1102 (18.14%)	4408 (18.14%)	
≥80	54 (0.89%)	216 (0.89%)	
Gender			1.0000
Male	3914 (64.42%)	15,656 (64.42%)	
Female	2162 (35.58%)	8648 (35.58%)	
Co-morbidities			
Hypertension	1597 (26.28%)	5362 (22.06%)	<0.0001
Diabetes mellitus	797 (13.12%)	2681 (11.03%)	<0.0001
Ischemic heart disease	592 (9.74%)	1812 (7.46%)	<0.0001
Hyperlipidemia	1403 (23.09%)	4443 (18.28%)	<0.0001
Heart failure	214 (3.52%)	645 (2.65%)	0.0003
Peripheral vascular disease	159 (2.62%)	479 (1.97%)	0.0017
Cerebrovascular disease	410 (6.75%)	1307 (5.38%)	<0.0001
Dementia	34 (0.56%)	152 (0.63%)	0.5563
Chronic pulmonary diseases	1664 (27.39%)	3833 (15.77%)	<0.0001
Rheumatic disease	138 (2.27%)	377 (1.55%)	0.0001
Peptic ulcer disease	1780 (29.3%)	5177 (21.3%)	<0.0001
Liver disease	1536 (25.28%)	4519 (18.59%)	<0.0001
Hemiplegia or paraplegia	41 (0.67%)	227 (0.93%)	0.0533
Keratopathy	424 (6.98%)	1277 (5.25%)	<0.0001
Uveitis	56 (0.92%)	158 (0.65%)	0.0236
Glaucoma	120 (1.97%)	335 (1.38%)	0.0006
AMD	51 (0.84%)	141 (0.58%)	0.0226

AMD = age-related macular degeneration.

**Table 2:** Incidence rate of dry eye disease in the study group.

Event	Study *n* = 6076	Control *n* = 24,304
Follow-up person months	307,691	1,260,167
New dry eye disease case	317	770
Incidence rate * (95% CI)	103.03 (92.29–115.02)	61.10 (56.93–65.57)
Crude Relative risk (95% CI)	1.685 (1.479–1.921)	Reference

* Incidence rate, per 100,000 person months. CI = confidential interval.

**Table 3:** Multiple Cox proportional hazard regression for estimation of adjusted hazard ratios on dry eye disease.

Variable	aHR (95% CI)
Surgery-indicated CRS	1.490 (1.303–1.702)
Age (Reference:40–59)	
<40	0.497 (0.420–0.588)
60–79	1.389 (1.194–1.616)
≥80	1.019 (0.548–1.894)
Gender (Reference: Female)	
Male	0.504 (0.447–0.569)
Co-morbidities	
Hypertension	0.994 (0.847–1.166)
Diabetes mellitus	1.016 (0.849–1.216)
Ischemic heart disease	0.980 (0.802–1.198)
Hyperlipidemia	1.143 (0.975–1.339)
Heart failure	0.786 (0.573–1.079)
Peripheral vascular disease	1.683 (1.278–2.217)
Cerebrovascular disease	1.028 (0.820–1.290)
Dementia	0.740 (0.363–1.505)
Chronic pulmonary diseases	1.221 (1.058–1.409)
Rheumatic disease	1.212 (0.876–1.677)
Peptic ulcer disease	1.547 (1.353–1.770)
Liver disease	1.191 (1.031–1.376)
Hemiplegia or paraplegia	0.892 (0.485–1.639)
Keratopathy	2.300 (1.907–2.773)
Uveitis	1.664 (1.049–2.639)
Glaucoma	2.132 (1.582–2.873)
AMD	1.620 (1.005–2.611)

CRS = chronic rhinosinusitis, aHR = adjusted hazard ratio, CI = confidential interval, AMD = age-related macular degeneration.

**Table 4:** The sensitivity analysis for the adjusted hazard ratio stratified by follow-up time of gender and age groups.

Subgroups	Incidence Rate (95% CI)		aHR (95% CI)
	Study	Control	
**Gender**			
Male	71.29 (60.55–83.94)	43.89 (39.58–48.67)	1.418 (1.165–1.726)
Female	163.63 (140.98–189.93)	93.18 (84.58–102.65)	1.580 (1.316–1.897)
*p* for interaction			0.6765
**Age**			
<40	163.63 (140.98–189.93)	93.18 (84.58–102.65)	1.368 (0.997-–1.878)
40–59	124.55 (106.97–145.02)	65.54 (59.12–72.65)	1.670 (1.383–2.018)
60–79	192.26 (156.89–235.59)	127.17 (112.43–143.85)	1.329 (1.042–1.694)
≥80	260.77 (97.87–694.80)	92.97 (44.32–195.02)	9.196 (1.153–73.357)
*p* for interaction			0.3170

CI = confidential interval, aHR = adjusted hazard ratio.
